# Supplementary material for: A transcriptome atlas of the mouse iris at single-cell resolution defines cell types and the genomic response to pupil dilation
Source: eLife. 2021 Nov 16;10:e73477. doi: 10.7554/eLife.73477 (PMC8594943; doi:10.7554/eLife.73477)
Supplement: Supplementary file 1. [file elife-73477-supp1.docx]

**Supplementary File 1.**

**Libraries**

library treatment nuclei mean number of transcripts per nucleus

JW02 control 1479 1883

JW03 constricted 4232 1628

JW04 dilated 7321 1790

JW13 constricted 1274 2256

JW15 constricted 3459 1589

JW16 dilated 3706 1619

JW17 control 6392 1632

JW18 control 6494 1681

total 34357

**Number of nuclei per cluster**

constricted control dilated total

CB 310 346 635 1291

CBE 247 373 675 1295

dilator 3376 4357 3873 11606

EC 51 149 73 273

IPE 2247 3719 2738 8704

leukocytes 92 193 102 387

sphincter 1 232 544 235 1011

sphincter 2 203 352 157 712

stroma 1 538 1292 485 2315

stroma 2 1669 3040 2054 6763

total 8965 14365 11027 34357
